# The effects of alteration in muscle activation on the iliotibial band during an exhaustive run

**DOI:** 10.1186/s13102-023-00709-0

**Published:** 2023-08-10

**Authors:** Shane Fei Chen, Yan Wang, Fangbo Bing, Ming Zhang

**Affiliations:** 1 Department of Biomedical Engineering, Faculty of Engineering, The Hong Kong Polytechnic University, Hong Kong, 999077 China; 2 Hong Kong Polytechnic University Shenzhen Research Institute, Shenzhen, 518057 China; 3 Research Institute for Sports Science and Technology, The Hong Kong Polytechnic University, Hong Kong, 999077 China

**Keywords:** Iliotibial band, Muscle activities, Exhaustive running, Knee stability

## Abstract

**Purpose:**

Long exhausted running causes pain at the lateral femoral epicondyle for some runners. The pain has been revealed to be related to the behavior of the iliotibial band (ITB) during running. The purpose of this study is to examine the effects of in-series musculature on the behavior of the ITB in healthy participants during an exhaustive run.

**Methods:**

Twenty-five healthy participants (15 males, 10 females) were recruited in the current study. All participants performed a 30-minute exhaustive run at a self-selected speed with laboratory-provided footwear. Muscle activities of ITB-related muscles including tensor fascia latae (TFL), gluteus maximus (Gmax), gluteus medius (Gmed), biceps femoris (BF), and vastus lateralis (VL) were recorded using surface electromyography (EMG).

**Results:**

Maximum amplitudes at the initial stage (the first minute), the mid stage (the 15-minute), and the end stage (the 30-minute) were compared during the exhaustive running. Significant decreases (*p* < 0.05) were observed in the maximum amplitudes of the TFL, Gmax, Gmed, and BF at the mid (decreased by ~ 15%) and end (decreased by ~ 30%) stages compared to the initial stage. The onset and the offset remained unaltered during the running (p ≥ 0.05).

**Conclusion:**

The behavior of the healthy ITB might be altered due to the activities of the in-series musculature. Excessive compression forces might be applied to the lateral femoral epicondyle from the ITB to provide stability for the knee joint during an exhaustive run. The findings could provide a basic understanding of the behavior of healthy ITB.

## Introduction

Iliotibial band syndrome (ITBS) is a prevalent overuse injury with a high incidence of 22.2% of all lower extremity injuries [1]. It takes up a high proportion of running-related injuries [2, 3]. Patients with ITBS feel pain after a few kilometers of running, and the pain in the lateral femoral epicondyle would intensify as they continue [4]. The pain often prohibits runners to perform sports activities temporarily or permanently in server cases [5]. One predominant etiology of ITBS is that excessive compression forces of the iliotibial band (ITB) applied to lateral femoral epicondyle result in it [6]. The knee compression forces achieved by the tensioning of the ITB were proposed to be directly influenced by running biomechanics [7].

The ITB is a thickening part of the superficial fascia with the majority insertion into the lateral tibial condyle [8]. In the proximal position, the ITB receives tension from the tensor fascia lata (TFL), gluteus maximus muscle (Gmax) [9], and gluteal aponeurosis over the gluteus medius muscle (Gmed). In the distal position, the ITB is distorted by the contraction of vastus lateralis muscle (VL) and biceps femoris muscle (BF) [10, 11]. Due to the complex anatomical structures, the ITB coordinates with these muscles to provide stability for knee and hip joints during running [12]. As the ITB fully inserts into the TFL and partly connects to Gmax, it receives tension from these two muscles to contribute to the anterolateral rotation stability for the knee joint. Additionally, a previous magnetic resonance image study [6] indicated that the tension in the ITB created by the muscles could produce compression in the tissues between the distal ITB and the lateral femoral epicondyle. Consequently, knee stability could be obtained from the compression forces applied to the lateral femoral epicondyle by the tensioning of the ITB [7]. The ITB was suggested to be one of the most important stabilizers of the knee joint, especially during high knee flexion angles [13].

Modified alteration in the force transmission from connected structures might lead to overuse injuries [14]. Force transmission in the ITB was mostly determined by its relevant muscles. These muscle activations might be modified during an exhaustive run [15, 16]. Additionally, passive loading in the tissues was revealed to increase during an exhaustive run, which might lead to a higher risk of injury [17]. Surface electromyography (EMG) was usually adopted to measure muscle activities [18, 19]. For this technique, the average amplitudes of the EMG signals could quantify the level of muscle activation for a given period without any invasion [20]. Some previous researchers have reported the associations between EMG amplitudes and injury risk indirectly [21]. The increase in the EMG amplitudes was associated with the increase in the musculotendon stiffness for major muscles of the lower limb [22]. Souza and Powers [23] adopted surface EMG to discuss the association between hip muscles and knee pain. Rabita et al. [24] analyzed the amplitudes of BF using surface EMG, which revealed that there was a significant decrease in the pre-activation phase during an exhaustive run. Another study examined the activities of Gmed and TFL during an exhaustive run, which indicated that the amplitudes of both the muscles would decrease significantly [25]. On the other hand, the identification of the timing including the onset and offset of the muscles could confirm the duration of muscles staying on. However, due to the artifacts and noise of surface EMG signals in running, the timing detection might be impaired. Teager-Kaiser energy operator (TKEO) could be applied to EMG signals to obtain more reliable results [26, 27]. The algorithm detected the first timing point exceeded a baseline as the onset timing for a specific muscle. The offset timing was set to be the first point below the threshold. Muscles begin to activate with an instantaneous augmentation in EMG signals, which could be detected by TKEO. Therefore, the current study adopted TKEO to obtain the temporal parameters.

Runners perform cyclic and repetitive movements [28]. Since the pain caused by ITBS appears after a period of running [29] but not at the beginning, the behavior of the ITB might be changed to cause an excessive and abnormal increase of compression forces between the ITB and the lateral femoral epicondyle. The activities of in-series musculature might be altered during an exhaustive run, which could change the behavior of the ITB. Few studies investigated the timing of the ITB-related muscles and amplitudes in different phases of the exhaustive run. Consequently, the current study hypothesizes that muscle activities would be altered, which modifies the behavior of the ITB during an exhaustive run. Additionally, we also hypothesized that the compression forces from the ITB applied to the lateral femoral epicondyle would increase due to the altered muscle activities under repetitive and cyclic running activities.

## Methodology

### Participants

A sample size was estimated using Gpower (G*power 3.0.10, Universität Düsseldorf, Germany) with a significant level of 0.05. The sample size in the current study met the requirement with a medium effect size of 0.8. Twenty-five healthy participants (15 males, and 10 females, 64.7 ± 11.7 kg, 170.9 ± 8.4 cm) were recruited from the Hong Kong Polytechnic University. The age of all the participants was between 20 and 30 years old and their BMI was between 19 and 24. Participants were excluded if they had musculoskeletal injuries in the preceding 12 months. Participants who have excessive foot pronation or supination were also excluded. The recruited participants run at least 10 miles a week. Informed written consents were signed by all participants before the experiment. The Human Subjects Ethics Sub-Committee of the Hong Kong Polytechnic University (Number: HSEARS20150121003) has approved this study.

### Experiment setup and data collection

A wireless EMG system DelSys Trigno (DelSys, Boston, MA) was used to record activities of TFL, Gmax, Gmed, BF, and VL at a sample rate of 2000 Hz. The EMG sensors have 8-mm Ag/AgCl with a 22 cm inter-electrode spacing. Bipolar differential surface electrodes (Ag/AgCl) were attached to the belly of each muscle with clean and shaven skin in the right leg. The direction of the electrode position was parrel to the muscle fiber as referred by ‘surface EMG for non-invasive assessment of muscles’ (SENIAM) which is a European project on surface EMG [30]. The SENIAM standards focus on surface EMG and recommend locations of electrodes for surface EMG. The EMG for TFL was located on the proximal 1/6 of the line from the anterior spinal iliac superior to the lateral femoral condyle. The EMG for Gmax was attached at the 1/2 line between the great trochanter and the sacral vertebrae and the EMG for Gmed was attached at the 1/2 line between the crista iliac and the trochanter. The placement of the EMG for VL was at the 2/3 on the line from the anterior spinal iliac superior to the lateral side of the patella. The EMG for BF was placed at 1/2 line between the ischial tuberosity and the lateral epicondyle of the tibia. Additionally, BF was assumed to have identical EMG activation patterns in the long and short heads [31]. All the electrodes were placed by the same research assistant to confirm the location consistency for all the muscles and participants.

All participants wore neutral, laboratory-provided sports shoes (ARHQ025-4, Li-Ning Inc., Beijing, China). Before the formal treadmill running test, a 5-minute warm-up was performed and then another 5 min was used to be familiar with the treadmill (Unisen Inc., Tustin, CA, USA) for participants. Then participants were required to run at their self-selected speeds to perform an exhaustive run on a treadmill for 30 min [32]. The running trial was continuous at this fixed speed. Raw surface EMG signals of the five muscles were recorded for 1 min at the 1-minute (initial stage), 15-minute (mid stage), and 30-minute (end stage) of the 30-minute exhaustive running trial.

### Data processing

The raw EMG signals were filtered with a 20 Hz fourth-order Butterworth high-pass filter to remove electrode artifacts firstly [18, 33]. Then, TKEO algorithm was performed [26, 27]. A high-pass filter with 10–20 Hz cutoff frequencies was applied to remove artifacts. The second-order bandpass Butterworth filter with 10 − 500 Hz was applied after full wave rectification to the EMG signals. Linear envelopes of EMG signals were created using a low-pass second-order Butterworth filter with a cut-off frequency of 6 Hz. An EMG profile for each muscle was represented by averaging linear envelops of ten consecutive cycles [34]. EMG data were normalized to the maximum amplitude at the initial running to perform comparisons.

Temporal parameters of surface EMG signals include the onset and offset of EMG bursts. For detecting muscle firing timing, a threshold was set to be 10% of the local peak of the maximum amplitude. The onset and offset points were determined by the intersection of the single envelop and the threshold [34]. The onset and offset timing were obtained by averaging ten consecutive cycles, and then represented as the percentage of the gait circle.

### Statistical analysis

SPSS Version 19.0 (IBM Corp., Armonk, NY, USA) was used to perform statistical analysis. The normal distribution of the variables of interest was examined using the Shapiro-Wilk test with *p* > 0.05. As some of the parameters revealed nonnormal distributions, a non-parametric Friedman Two-Way Analysis of Variance (ANOVA) was adopted to verify the differences between different conditions. The peak amplitudes of TFL, Gmax, Gmed, BF, and VL at different stages were used to perform the statistical analyses. If there are significant differences, the post-hoc pairwise *t*-tests with Bonferroni method were performed. The significant level *p*-value was set at 0.05.

## Results

**Fig. 1:**
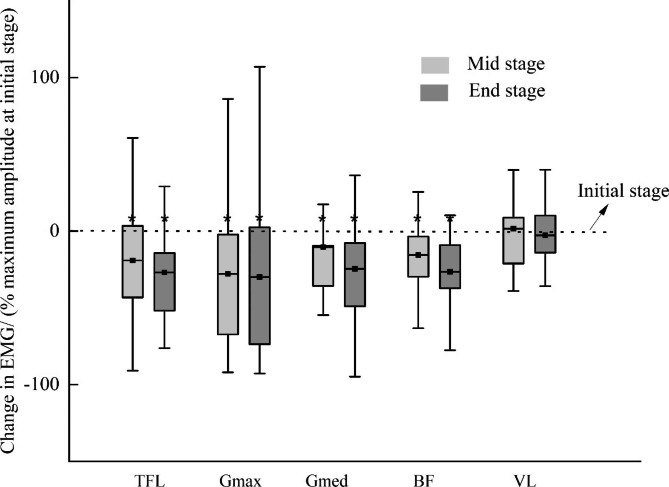
Change in EMG amplitude for TFL, Gmax, Gmed, BF, and VL. The statistical results were detected using the maximal amplitudes for each muscle ⁎ Significant difference between different stages at *p*-value < 0.05. Tensor fascia latae = TFL; gluteus maximus = Gmax; gluteus medius = Gmed; biceps femoris = BF; vastus lateralis = VL.

Figure 1 shows the changes in peak amplitudes for each muscle at the mid and end stages when compared to the peak amplitude at the initial stage. The statistical results were examined using the peak amplitudes at the initial, mid, and end stages. Significant decreases were observed in the maximum amplitudes of TFL, Gmax, Gmed, and BF during an exhaustive run. The activation of these four muscles decreased significantly at the mid and end stages when compared to the initial stage whereas no significant differences were revealed between the mid stage and the end stage. Furthermore, as shown in Fig. 1, the maximum amplitude of Gmax in the mid stage decreased to 72.09% of the maximum amplitude in the initial stage whereas the activation in TFL, Gmed, and BF remained at over 80% of the activation of the initial stage, which might indicate a rapid decrease in the activation of Gmax.

**Fig. 2:**
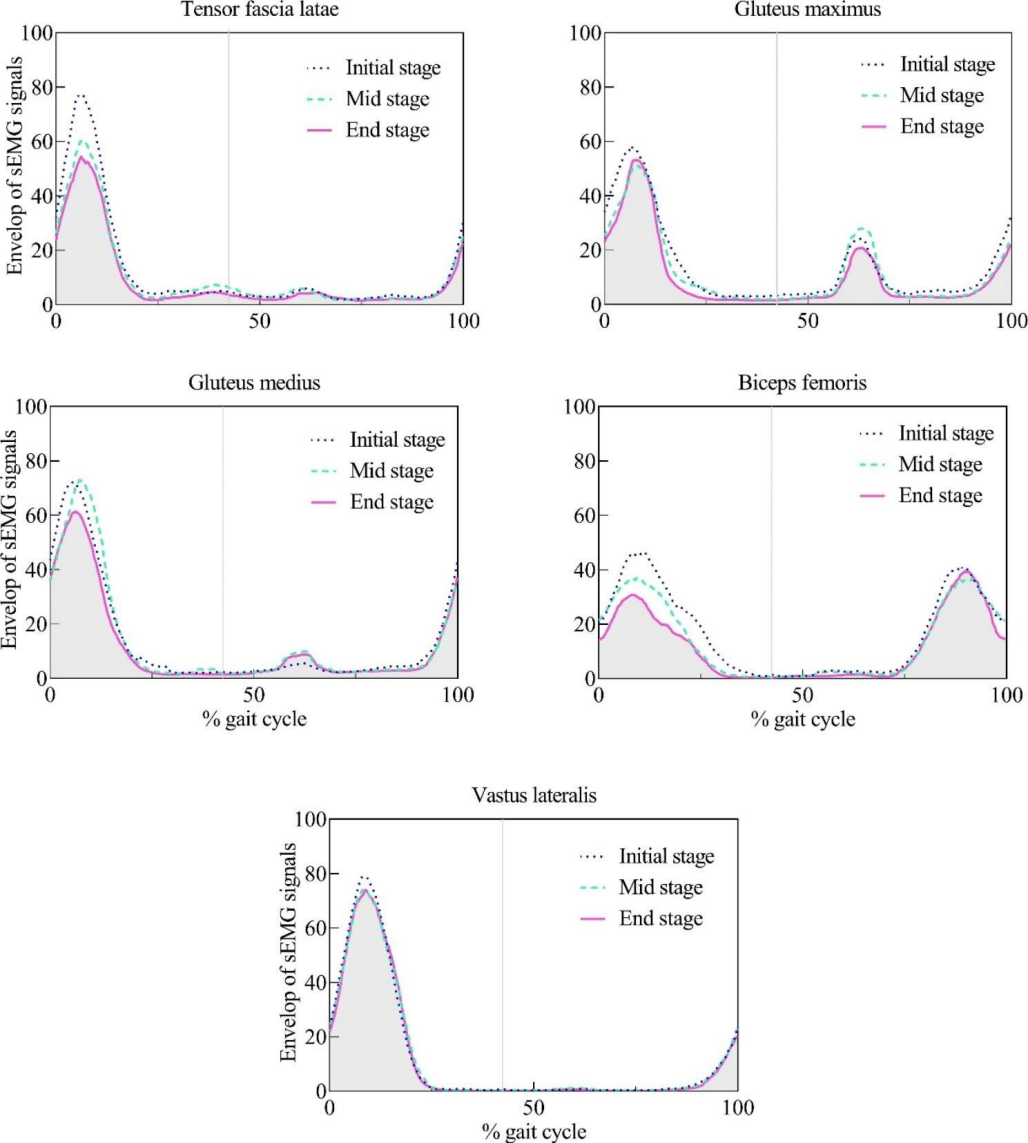
EMG activity patterns during an exhaustive run Heel strike is at 0% and 100% of the stride.

**Fig. 3:**
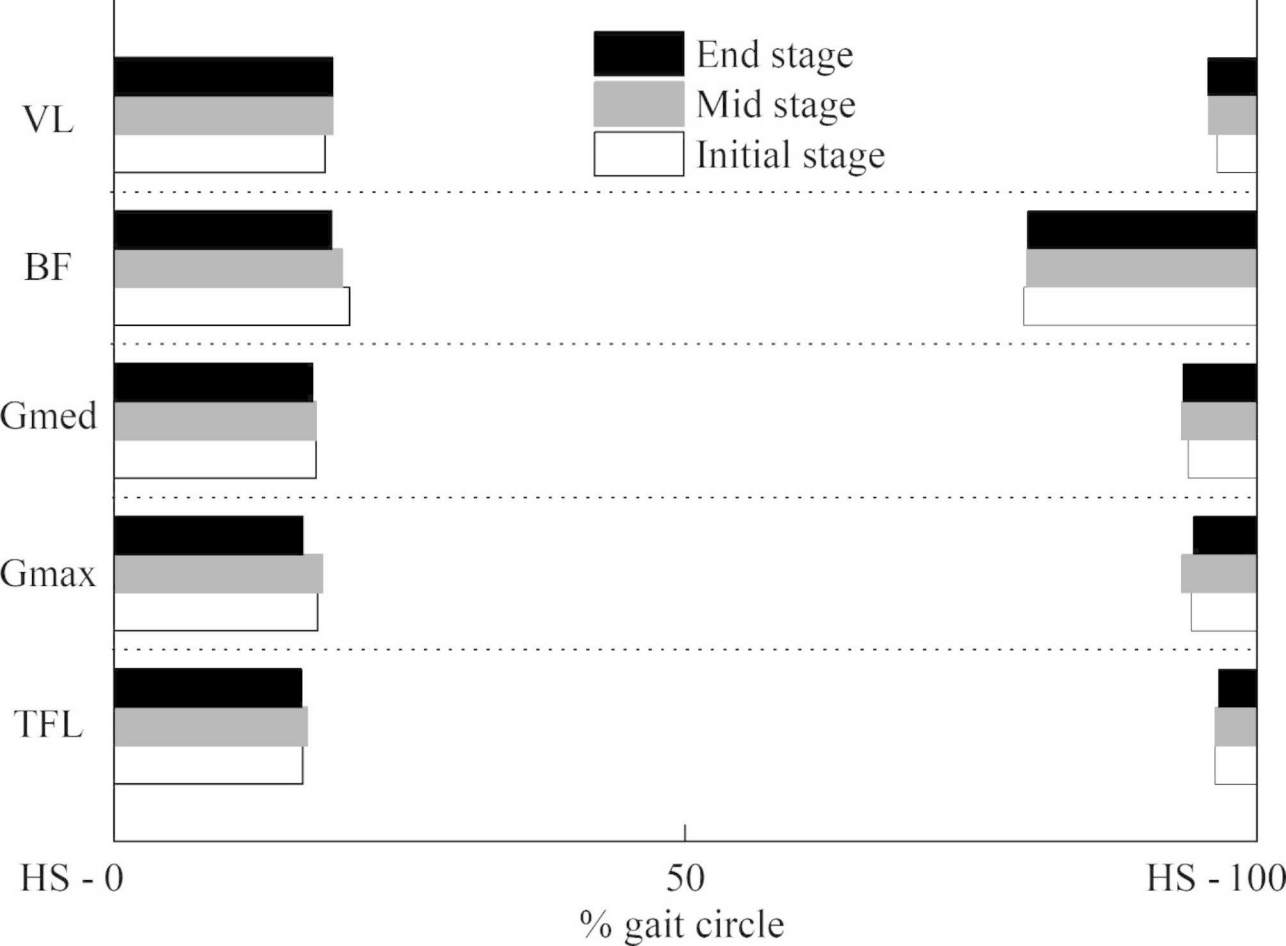
Relative muscle timing. Each bar represents the muscle staying on based on the detecting threshold Tensor fascia latae = TFL; gluteus maximus = Gmax; gluteus medius = Gmed; biceps femoris = BF; vastus lateralis = VL. HS: heel strike.

The EMG activation patterns were shown in Fig. 2. The onset and offset timing of the TFL, Gmax, Gmed, BF, and VL in different stages were listed in Table 1. Only the onset in the late swing phase and the onset in the stance phase were compared. The short activation during the swing phase such as Gmax was not included in the current study. No differences were observed in the onset and offset between the initial and mid or end stages. The firing timing of the muscles was indicated to be stable during the whole exhaustive running. Figure 3 shows the relative muscle timing based on the detecting threshold.

**Table 1:** Muscle timing at different stages (mean ± SD)

Variable		Initial stage (% gait circle)	Mid stage (% gait circle)	End stage (% gait circle)	*P*
TFL	onset	96.35 ± 3.75	96.37 ± 3.24	96.65 ± 3.73	0.529
	offset	16.55 ± 3.97	16.92 ± 3.81	16.39 ± 3.66	0.424
Gmax	onset	94.29 ± 5.81	93.43 ± 6.01	94.46 ± 6.48	0.435
	offset	17.81 ± 5.19	18.24 ± 4.49	16.52 ± 4.01	0.061
Gmed	onset	94.01 ± 3.57	93.41 ± 4.28	93.56 ± 3.27	0.157
	offset	17.68 ± 4.21	17.71 ± 3.91	17.39 ± 4.06	0.237
BF	onset	79.62 ± 8.41	79.88 ± 8.35	79.91 ± 8.26	0.876
	offset	20.62 ± 9.49	19.95 ± 9.75	19.08 ± 8.64	0.585
VL	onset	96.55 ± 4.00	95.76 ± 3.95	95.73 ± 3.44	0.131
	offset	18.45 ± 3.34	19.15 ± 3.33	19.16 ± 2.75	0.056

⁎ Significant difference between stages at *P* value < 0.05.

Tensor fascia latae = TFL; gluteus maximus = Gmax; gluteus medius = Gmed; biceps femoris = BF; vastus lateralis = VL.

## Discussion

The study aimed to examine the effects of alteration in muscle activities on the behavioral adaption of the ITB during running. The activation of TFL, Gmax, Gmed, and BF decreased significantly in the mid and end stages during an exhaustive run. Furthermore, the maximum amplitudes of Gmax demonstrated a rapid decrease in the mid stage. The firing timing of muscles revealed no significant differences during the whole running stages. Changes in the activations of the in-series musculature of the ITB might alter its behavior during different stages of an exhaustive run.

The ITB receives direct muscle forces from TFL and Gmax due to the fully and partly anatomical insertions. The changes in myofascial force transmission strategy were associated with the development of overuse injuries in running as the structural linkage of the muscles with connective tissues [14]. Furthermore, long exhaustive running could lead to an unfamiliar and compensating running pattern [35]. Thus, the behavior of the ITB was postulated to be altered during an exhaustive run. Compared to the initial stage of the running, TFL and Gmax revealed a significantly decreased activation in the mid and end stages, especially for a rapid decrease in the mid stage for Gmax. The decrease in the activation of the TFL and Gmax might lead to a changing behavior of the ITB. Hutchinson et al. [7] developed a simplified free-body diagram to illustrate the tension in the ITB is associated with knee compression forces. The underactivity of the gluteal muscles and the overactivity of TFL might lead to an increase in tension in the ITB [32]. Though both the TFL and Gmax tended to activate weakly as the running duration increased, the Gmax revealed a rapid decrease with 27.91% of the maximum amplitude at the initial stage in the mid stage compared to the decrease of TFL with 19.17%, which might lead to a dysfunction of overactivity in TFL and underactivity in Gmax compared to the initial stage. Additionally, the underactivity of Gmed in the mid and end stages would result in a compensatory activation of TFL, which would cause higher tension force in the ITB [5]. Consequently, the increase of tension in the ITB would cause a higher compression force between the ITB and lateral femoral epicondyle. Bertelsen et al. [36] constructed a conceptual model to understand strain-related overuse injuries that repetitive loading of tissues could cause microdamage accumulation. The overuse injuries could be contributed by microdamage without an appropriate adaptation. The weakness of the hip abductor might lead to a large hip adduction angle that could cause an increase in the strain of the ITB during the stance phase of running [37]. The alteration in the activation patterns of TFL and Gmax might lead to excessive strain in the ITB. Through the force transmission from TFL and Gmax, the ITB also contributes to the stabilization of the knee joints in some way.

A deep capsule-osseous layer as the part of distal ITB originates from the proximity to the lateral gastrocnemius tubercle and attaches to the lateral tibial tubercle on the anterolateral aspect of the proximal tibia [8, 38] that stabilizes the knee joint. The ITB was demonstrated to be the secondary stabilizer for tibia rotation except for the anterior cruciate ligament [13], which plays an important role at high knee flexion angles where the anterior cruciate ligament functions less. Hip muscles connected with the ITB assist to balance the biomechanical forces in the human body. At heel strike, the trunk leans to the same side of the strike to absorb shock. The stabilization of the lateral trunk flexion is balanced by the contraction of the hip muscles. The stabilization of the knee joint could be achieved from the compression force between the ITB and the lateral femur through tensioning the ITB [7]. The running duration was related to the decrease in joint laxity due to muscular activation [39]. The maximum of the TFL, Gmax, and Gmed decreased significantly in the mid and end stages during the exhaustive running, which might indicate a weak ability to control the knee joints. Therefore, extra compression force applied to the lateral femoral epicondyle from the ITB might be a compensatory running pattern to provide more stabilization for the knee joint during an exhaustive run.

VL is another ITB-tensing muscle, which subsequently contributes to the lateral stabilization of the pelvis due to its ‘hydraulic amplifier’ action on the fascia latae [40]. The VL acts as an adjustable lever arm that could increase the distance between the ITB and femoral shaft when it contracts, which thus results in the increase of tension in the ITB. The maximum amplitudes of VL remained to be the same level during the exhaustive running, which revealed the same results in a previous study [24]. The results could be due to the compensation strategy of running [24]. On the contrary, the maximum amplitude of BF demonstrated a significant decrease in the mid and end stages compared to the initial stage during the exhaustive running. BF acts to be a lateral stability structure of the knee joint, which inserts into the posterior edge of the ITB [41, 42]. The contraction of the BF could decrease the internal rotation of the tibia, which could lead to less tension in the ITB [43]. The activation of the BF tended to decrease significantly, which might lead to less limitation of the internal rotation of the tibia. Consequently, extra traction in the ITB could potentially increase to provide stability for the knee joint during an exhaustive run.

The amplitudes of muscles describe the gross input for the given tasks. However, the timing parameters including the onset and offset of a specific muscle could reflect when the muscle turns on and off. In the current study, both the onset and offset of TFL, Gmax, Gmed, BF, and VL revealed no significant differences during the whole exhaustive running. The results were consistent with the previous findings [25] that indicated no significant differences in the onset of TFL, Gmax, and Gmed during an exhaustive run. Alteration in the muscle activation timing was associated with the increase of hip adduction angles, which thus might lead to compression at the femoral epicondyle [44]. However, the results from the current study did not reveal that the timing alteration would appear in healthy ITB during an exhaustive run. Future studies could prolong the duration and intensity of the running to examine the activation timing of the muscles.

ITBS is one common overuse injury that could be caused by multifactor. The activation of muscles related to ITB would change during an exhaustive run, which might indicate an altered force transmission through these muscles. The changed running biomechanics and altered force transmission from the altered muscular activities during an exhaustive run might have influences on the mechanical performance of ITB. Thus, treatments for ITBS should take muscle strength into consideration.

## Limitations

The current study had some limitations. Some differences may exist between overground running and treadmill running. Participants could not alter their running speed to mitigate muscle activation. Regardless of the effort to skin preparation and electrode placements, movement artifacts and noise still need to be considered for surface EMG. Additionally, participants were provided laboratory footwear to eliminate footwear bias. However, some participants might not be accustomed to it. Runners were required to perform the exhaustive running on a treadmill at a self-selected speed, leading to speed differences between females and males. It should also be noted that different leg muscle fitness of the participants might cause bias. Future studies could examine the differences in muscle activities between males and females.

## Conclusion

The current study examined the effects of the alteration of in-series muscles on the behavior of the ITB using physiological parameters during an exhaustive run. A rapid decrease in the maximum amplitudes of Gmax might lead to a relative underactivity of Gmax, which could increase the tension in the ITB. The activation of the BF also decreased significantly, which could also potentially augment the ITB tension. The tension increase in the ITB might predispose to ITBS. Strengthening of hip muscles is suggested to offer better prevention of such overuse injuries. The results of this study would provide a basic understanding of the behavior of the healthy ITB, which might give some insights into the etiology of ITB-related pathologies, such as ITBS.

## Data Availability

The datasets during the current study are available from the corresponding author on reasonable request.

## References

[1] Hamstra-Wright KL, Jones MW, Courtney CA, Maiguel D, Ferber R (2020). Effects of iliotibial band syndrome on pain sensitivity and gait kinematics in female runners: a preliminary study. Clin Biomech (Bristol Avon).

[2] Taunton JE, Ryan MB, Clement DB, McKenzie DC, Lloyd-Smith DR (2002). Zumbo. BD. A retrospective case-control analysis of 2002 running injuries. Br J Sports Med.

[3] Kakouris N, Yener N, Fong DTP (2021). A systematic review of running-related musculoskeletal injuries in runners. J Sport Health Sci.

[4] Noble CA (1980). Iliotibial band friction syndrome in runners. Am J Sports Med.

[5] Friede MC, Innerhofer G, Fink C, Alegre LM, Csapo R (2021). Conservative treatment of iliotibial band syndrome in runners: are we targeting the right goals?. Phys Ther Sport.

[6] Fairclough J, Hayashi K, Toumi H, Lyons K, Bydder G, Phillips N (2006). The functional anatomy of the iliotibial band during flexion and extension of the knee: implications for understanding iliotibial band syndrome. J Anat.

[7] Hutchinson LA, Lichtwark GA, Willy RW, Kelly LA. The Iliotibial Band: a Complex structure with versatile functions. Sports Med. 2022. 10.1007/s40279-021-01634-3 PMC9023415 35072941

[8] Godin JA, Chahla J, Moatshe G, Kruckeberg BM, Muckenhirn KJ, Vap AR (2017). A comprehensive reanalysis of the Distal Iliotibial Band: quantitative anatomy, radiographic markers, and Biomechanical Properties. Am J Sports Med.

[9] Flato R, Passanante GJ, Skalski MR, Patel DB, White EA, Matcuk GR (2017). Jr. The iliotibial tract: imaging, anatomy, injuries, and other pathology. Skeletal Radiol.

[10] Besomi M, Salomoni SE, Cruz-Montecinos C, Stecco C, Vicenzino B, Hodges PW. Distinct displacement of the superficial and deep fascial layers of the iliotibial band during a weight shift task in runners: an exploratory study. J Anat. 2021. 10.1111/joa.13575 PMC8819045 34697798

[11] Birnbaum K, Siebert CH, Pandorf T, Schopphoff E, Prescher A, Niethard FU (2004). Anatomical and biomechanical investigations of the iliotibial tract. Surg Radiol Anat.

[12] Foch E, Aubol K, Milner CE (2020). Relationship between iliotibial band syndrome and hip neuromechanics in women runners. Gait Posture.

[13] Kaplan DJ, Jazrawi LM (2018). Secondary stabilizers of tibial rotation in the Intact and Anterior Cruciate Ligament deficient knee. Clin Sports Med.

[14] Wilke J, Vleeming A, Wearing S (2019). Overuse Injury: the result of pathologically altered Myofascial Force Transmission?. Exerc Sport Sci Rev.

[15] Brown AM, Zifchock RA, Hillstrom HJ, Song J, Tucker CA (2016). The effects of fatigue on lower extremity kinematics, kinetics and joint coupling in symptomatic female runners with iliotibial band syndrome. Clin Biomech (Bristol Avon).

[16] Cowley JC, Gates DH (2017). Proximal and distal muscle fatigue differentially affect movement coordination. PLoS ONE.

[17] Debenham J, Travers M, Gibson W, Campbell A, Allison G (2016). Eccentric fatigue modulates Stretch-shortening cycle Effectiveness–A possible role in Lower Limb Overuse Injuries. Int J Sports Med.

[18] Gazendam MG, Hof AL (2007). Averaged EMG profiles in jogging and running at different speeds. Gait Posture.

[19] Schache AG, Dorn TW, Williams GP, Brown NA, Pandy MG (2014). Lower-limb muscular strategies for increasing running speed. J Orthop Sports Phys Ther.

[20] Hsu WC, Tseng LW, Chen FC, Wang LC, Yang WW, Lin YJ (2020). Effects of compression garments on surface EMG and physiological responses during and after distance running. J Sport Health Sci.

[21] Hunter I, Seeley MK, Hopkins JT, Carr C, Franson JJ (2014). EMG activity during positive-pressure treadmill running. J Electromyogr Kinesiol.

[22] Kyrolainen H, Avela J, Komi PV (2005). Changes in muscle activity with increasing running speed. J Sports Sci.

[23] Souza RB, Powers CM (2009). Differences in hip kinematics, muscle strength, and muscle activation between subjects with and without patellofemoral pain. J Orthop Sports Phys Ther.

[24] Rabita G, Couturier A, Dorel S, Hausswirth C, Le Meur Y (2013). Changes in spring-mass behavior and muscle activity during an exhaustive run at VO2max. J Biomech.

[25] Brown AM, Zifchock RA, Lenhoff M, Song J, Hillstrom HJ (2019). Hip muscle response to a fatiguing run in females with iliotibial band syndrome. Hum Mov Sci.

[26] Li X, Zhou P, Aruin AS (2007). Teager-Kaiser energy operation of surface EMG improves muscle activity onset detection. Ann Biomed Eng.

[27] Solnik S, Rider P, Steinweg K, DeVita P, Hortobagyi T (2010). Teager-Kaiser energy operator signal conditioning improves EMG onset detection. Eur J Appl Physiol.

[28] Gaudreault N, Boyer-Richard É, Fede C, Fan C, Macchi V, De Caro R et al. Static and dynamic Ultrasound Imaging of the Iliotibial Band/Fascia Lata: brief review of current literature and gaps in knowledge. Curr Radiol Rep. 2018;6(10).

[29] Baker RL, Souza RB, Fredericson M (2011). Iliotibial band syndrome: soft tissue and biomechanical factors in evaluation and treatment. PM R.

[30] Hermens HJ, Freriks B, Merletti R, Stegeman D, Blok J, Rau G (1999). European recommendations for Surface ElectroMyoGraphy. Roessingh Res Dev.

[31] Chen TL, Wong DW, Xu Z, Tan Q, Wang Y, Luximon A (2018). Lower limb muscle co-contraction and joint loading of flip-flops walking in male wearers. PLoS ONE.

[32] Besomi M, Maclachlan L, Mellor R, Vicenzino B, Hodges PW (2020). Tensor Fascia Latae muscle structure and activation in individuals with Lower Limb Musculoskeletal Conditions: a systematic review and Meta-analysis. Sports Med.

[33] Baker RL, Souza RB, Rauh MJ, Fredericson M, Rosenthal MD (2018). Differences in knee and hip adduction and hip muscle activation in runners with and without Iliotibial Band Syndrome. PM R.

[34] Hug F (2011). Can muscle coordination be precisely studied by surface electromyography?. J Electromyogr Kinesiol.

[35] Van Wilgen CP, Verhagen EA (2012). A qualitative study on overuse injuries: the beliefs of athletes and coaches. J Sci Med Sport.

[36] Bertelsen ML, Hulme A, Petersen J, Brund RK, Sorensen H, Finch CF (2017). A framework for the etiology of running-related injuries. Scand J Med Sci Sports.

[37] Noehren B, Davis I, Hamill J (2007). ASB clinical biomechanics award winner 2006 prospective study of the biomechanical factors associated with iliotibial band syndrome. Clin Biomech (Bristol Avon).

[38] Runer A, Birkmaier S, Pamminger M, Reider S, Herbst E, Kunzel KH (2016). The anterolateral ligament of the knee: a dissection study. Knee.

[39] Tsai LC, Sigward SM, Pollard CD, Fletcher MJ, Powers CM (2009). Effects of fatigue and recovery on knee mechanics during side-step cutting. Med Sci Sports Exerc.

[40] Grimaldi A (2011). Assessing lateral stability of the hip and pelvis. Man Ther.

[41] Vieira EL, Vieira EA, da Silva RT, Berlfein PA, Abdalla RJ, Cohen M (2007). An anatomic study of the iliotibial tract. Arthroscopy.

[42] Whiteside LA, Roy ME (2009). Anatomy, function, and surgical access of the iliotibial band in total knee arthroplasty. J Bone Joint Surg Am.

[43] Kwak SD, Ahmad CS, Gardner TR, Grelsamer RP, Henry JH, Blankevoort L (2000). Hamstrings and iliotibial band forces affect knee kinematics and contact pattern. J Orthop Res.

[44] Fairclough J, Hayashi K, Toumi H, Lyons K, Bydder G, Phillips N (2007). Is iliotibial band syndrome really a friction syndrome?. J Sci Med Sport.

